# Relating protease inhibitor resistance at time of virological failure with drug exposure

**DOI:** 10.1186/1758-2652-13-S4-P118

**Published:** 2010-11-08

**Authors:** M Van Luin, WP Bannister, R Paredes, AN Phillips, J Bruun, J Van Lunzen, O Kirk, A d'Arminio Monforte, A Cozzi-Lepri, DM Burger

**Affiliations:** 1 Radboud University Nijmegen Medical Centre, Department of Clinical Pharmacy, Nijmegen, Netherlands; 2 University College London Medical School, London, UK; 3 Hospital Universitari Germans Trias i Pujol, Universitat Autònoma de Barcelona, Badalona, Spain; 4 Ullevål Hospital, Oslo, Norway; 5 University Medical Center Hamburg-Eppendorf, Hamburg, Germany; 6 Copenhagen HIV Programme, Panum Institute, Copenhagen, Denmark; 7 Istituto Di Clinica Malattie Infettive e Tropicale, Milan, Italy

## Background

The absence of detectable HIV resistance after treatment failure may result from non-adherence, especially for drugs such as ritonavir-boosted PIs (PI/r) for which minimum adherence may be sufficient to achieve suppression. This analysis aimed to investigate the association between adherence, indicated by a detectable PI plasma concentration, and development of PI resistance in patients presenting with virological failure of a PI/r regimen.

## Methods

Patients were included if they had virologically failed a PI/r, defined as a viral load (VL) >1000 copies/mL after ≥4 months continuous exposure to a PI/r and with a plasma sample available within 1 month of the estimated VL failure date. Samples were analysed for PI levels by a validated reversed-phase HPLC method; an undetectable PI level was defined as below the PI-specific lower limit of detection. Genotypic sequencing was also carried out retrospectively on the identified sample for those with no previous PI failure and PI resistance was defined as the presence of ≥1 major PI mutation (IAS-USA). Logistic regression was used to assess risk factors for an undetectable PI level and for detection of PI resistance at VL failure using exact methods for small datasets.

## Results

85 patients were included. PI/r regimens were started in Sept 2002 (median) with VL failure occurring a median time of 17 months later. At time of starting the PI/r (baseline), 57% were ARV-naïve, median CD4 count was 217 cells/mm^3^ and median VL was 4.8 log_10_copies/mL. 43 patients (51%) had an undetectable PI level at time of VL failure and were similar to those with detectable levels in terms of demographics, ARV history and previous VL failure. However, injecting drug use was associated with a greater risk of undetectable PI level (univariate odds ratio (OR) IDU vs. not: 3.7; 95% CI: 1.1-12.5; p=0.038).

44 (52%) of the 85 patients were successfully tested for resistance and had no previous PI failure. Those with undetectable PI levels were significantly less likely to have PI resistance (0% of 24 patients, 95% CI: 0-14%) than those with detectable levels (25% of 20, 95% CI: 9-49%), exact median unbiased estimate of OR: 0.1; p=0.029. Baseline VL, CD4 count, demographic and ARV-related variables were not associated with PI resistance due to limited power in this dataset.

## Conclusions

Non-adherence to a PI/r regimen, as measured by an undetectable PI level is linked to a lower rate of detection of PI resistance at time of VL failure.

